# Multiple Myeloma With Leptomeningeal Involvement: A Study of Three Cases Exploring Diagnosis and Treatment Challenges

**DOI:** 10.7759/cureus.81602

**Published:** 2025-04-02

**Authors:** Jamil Abou Issa, Urwat Vusqa, Eric M Behling, Tulin Budak-Alpdogan, Sushil Ghimire

**Affiliations:** 1 Internal Medicine, Cooper University Hospital, Camden, USA; 2 Hematology and Medical Oncology, Cooper University Hospital, Camden, USA; 3 Pathology, Cooper University Hospital, Camden, USA; 4 Hematology and Oncology, Cooper University Hospital, Camden, USA

**Keywords:** leptomeningeal involvement, mm cns, multiple myeloma, multiple myeloma with cns involvement, multiple myeloma with extramedullary disease

## Abstract

Central nervous system (CNS) involvement in multiple myeloma (MM) is a rare but serious complication that poses significant diagnostic and therapeutic challenges. This article presents three cases of leptomeningeal involvement in patients with MM, highlighting the diverse clinical presentations, diagnostic approaches, and treatment strategies employed. The first case is a 54-year-old female who, after initial treatment and autologous hematopoietic stem cell transplant, developed CNS disease. Similarly, a 73-year-old female developed leptomeningeal involvement with progressive neurological symptoms. The third case describes a 63-year-old female with immunoglobulin (Ig)A lambda MM who developed CNS disease after treatment with daratumumab and radiation. In all three cases, leptomeningeal enhancement and atypical plasma cells were identified in cerebrospinal fluid (CSF), with treatment strategies including intrathecal chemotherapy, systemic therapy, radiation, and stem cell transplantation. Despite aggressive management, including novel agents and supportive care, all patients had poor outcomes, with two transitioning to hospice care. The article reviews the limited literature on CNS-MM, noting the lack of standardized treatment protocols and the need for further research. As the survival of MM patients improves, the incidence of CNS involvement is expected to rise, making the development of targeted therapies essential. These cases underscore the urgent need for further investigation into novel treatment options and the importance of early diagnosis and comprehensive management of CNS-MM.

## Introduction

Multiple Myeloma (MM) with extramedullary disease (EMD) is defined as the presence of malignant plasma cells in soft tissues independent of the bone marrow microenvironment [1]. This includes tumors growing in the skin, lymph nodes, abdominal organs, respiratory system, and the central nervous system (CNS). The incidence of MM with EMD is around 0.5-4.8% at diagnosis, with skin and soft tissue being the most commonly involved organs. The incidence rate at the time of relapse changes to 3.4 to 14% [1,2]. Although a rare entity, the incidence of EMD has increased over the last few decades. This trend may be explained by the improvement in the overall survival (OS) of MM patients as more treatment options become available; this allows the remaining drug-resistant tumor cells to divide, proliferate, and develop further mutations [3]. MM with CNS involvement (CNS-MM) is defined as malignant cell infiltration of the CNS, meninges, and cerebrospinal fluid. The diagnosis is made by demonstrating evidence of plasma cell monoclonality in cerebrospinal fluid (CSF) [4]. Leptomeningeal involvement in patients with MM is one of the rarest presentations of EMDs. Its occurrence is extremely rare at initial MM diagnosis and makes up 20% of EMD relapses [3]. It is important to note that data on CNS-MM, including diagnosis, treatment, and prognosis, is sparse, and management of this entity is mostly based on single cases and retrospective studies due to the lack of clinical trials [5]. In this article, we present three interesting cases of leptomeningeal involvement in patients with multiple myeloma (MM), CARE checklist [6]. These three cases had different courses, prognostic factors, and outcomes, highlighting the heterogeneity of CNS-MM. Despite the rarity of this complication, each case contributes valuable insights to the scarce literature on CNS involvement in multiple myeloma.

## Case presentation

Case one

A 54-year-old female was initially referred to the hematology and oncology department due to concerns of monoclonal gammopathy. Her laboratory results showed an M-Spike of 3.5 g/dl and immunoglobulin (Ig)G kappa light chain restricted monoclonal gammopathy on immunofixation (IFE). She also reported neck pain, right-sided mandibular pain, and perioral numbness. Other relevant laboratory values are included in Table 1.

**Table 1:** Laboratory values for three cases in this study

Parameter	Normal Range	Patient 1	Patient 2	Patient 3
Hemoglobin	12.0-16.0 g/dl	12.4 g/dl	10.4 g/dl	10.2 g/dl
Creatinine	0.6-1.1 mg/dl	0.85 mg/dl	0.94 mg/dl	0.85 mg/dl
Calcium	8.5-10.5 mg/dl	9.3 mg/dl	10.3 mg/dl	10.9 mg/dl
Beta2-microglobulin	0.8-2.0 mg/dl	2.3 mg/dl	9.93 mg/dl	3.86 mg/dl
Lactate dehydrogenase	125-220 U/L	272 U/L	327 U/L	145 U/L
Albumin	3.5-5.0 g/dl	3.9 g/dl	3.0 g/dl	4.4 g/dl

**Figure 1:**
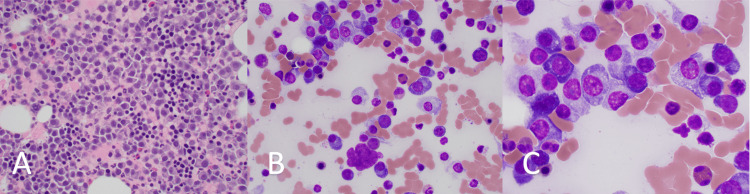
Bone marrow morphology showing bone marrow effacement with plasma cells with plasmablastoid features Panels A-C: Bone marrow showing effacement with plasma cells exhibiting plasmablastoid features at low, intermediate, and high magnifications, respectively.

A skeletal survey revealed lesions in the left proximal humerus and right femur. Concerned about her neck pain, an MRI of the neck, thoracic, and lumbar spine was performed, which showed a right mandibular mass, a mass in the skull base extending into the sphenoid sinus, and abnormal infiltrative lesions in the thoracic and lumbar marrow. A bone marrow biopsy was then conducted, revealing hypercellular marrow with marked atypical plasmacytosis (45-50%), consistent with bone marrow involvement by plasma cells with plasmablastic features, as seen in Figure 1. Flow cytometry showed CD45-/CD138++ plasma cells accounting for 11% of all analyzed cells, with cytoplasmic kappa light chain restriction. Cytogenetic analysis revealed a complex karyotype, with FISH results summarizedin Table 2.

**Table 2:** Clinical characteristics and treatment utilized Ig: Immunoglobulin, R-ISS: Revised International Staging System, FISH Test: Fluorescence in situ hybridization test, MM: Multiple myeloma, CNS: Central nervous system, EMD: Extramedullary disease, VDR: Volumetric diffusive ventilator, alloHSCT: Allogeneic hematopoietic stem cell transplantation, autoSCT: Autologous stem cell transplant, Dara-RVD: Daratumumab, lenalidomide, bortezomib, and dexamethasone combination.

	Patient 1	Patient 2	Patient 3
Age	54	73	63
Gender	F	F	F
MM subtype	IgG kappa light chain	IgA kappa light chain	IgA lamda light chain
Stage R-ISS	II	III	II
Bone marrow FISH	t(4;14), 1q gain, +trisomy 9 and 13	t(4;14), 1q gain, trisomy 9,7 and 13	t(11;14)
Adverse features	Complex karyotype, plasmablastic features, EMD	Complex cytogenetics, EMD, plasmablastic features	None
Time from MM diagnosis to CNS disease	13 months	32 months	10 months
Treatment prior to CNS disease	Radiation, VDR, carbo/lenalidomide/dex, alloHSCT	VDR, autoSCT, radiation	Dara-RVD, radiation
Treatment of CNS disease	Partial craniotomy, cranial and spinal radiation. Transitioned to hospice.	Hospice	IT chemotherapy, Pomalidomide/dexamethasone/venetoclax, switched to daratumumab and pomalidomide, Auto-HSCT with thiotepa and melphalan conditioning, switched to carfilzomib and venetoclax

The patient’s initial treatment included radiation to the right jaw, cervical spine, and right cavernous sinus for extramedullary lesions. Her systemic therapy consisted of four cycles of bortezomib, lenalidomide, and dexamethasone (VDR). Despite this, the disease progressed, with new lesions appearing in the left mandible, right rib, thoracic vertebrae, and right femur. Radiation was then administered to her right rib and femur. Her systemic regimen was switched to carfilzomib, lenalidomide, and dexamethasone for a total of three cycles. Approximately one year after her initial diagnosis, she underwent a two-step haploidentical allogeneic hematopoietic stem cell transplant (allo-HSCT), following a conditioning regimen of fludarabine, busulfan, and cyclophosphamide.

**Figure 2:**
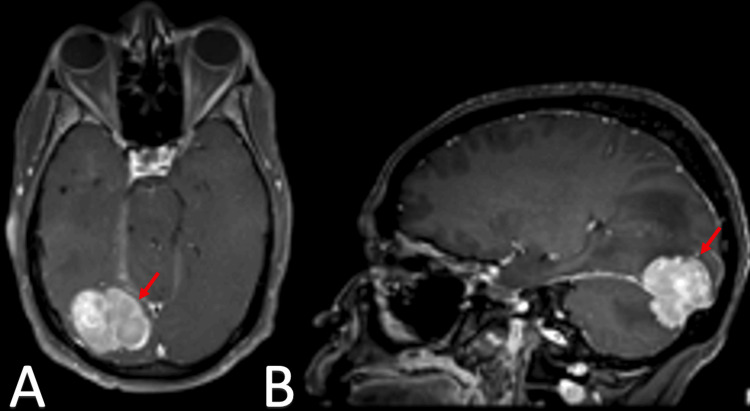
MRI imaging of extra-axial mass in the right occipital lobe Panel A: Axial T2-weighted MRI image showing a new extra-axial mass in the right occipital lobe, extending toward the cerebellum. Panel B: Sagittal T2-weighted MRI image of the same patient, highlighting the continuity of the extra-axial mass from the right occipital lobe to the cerebellum.

Three months post-transplant, the patient was hospitalized for intractable nausea, vomiting, and a significant 30-pound weight loss. A brain MRI showed a new extra-axial mass in the right occipital lobe extending to the cerebellum, as shown in Figure 2. She underwent a right craniotomy with partial resection of the brain lesion. Pathology confirmed CD138-positive cells, consistent with plasmablastic plasmacytoma. Following the surgery, she received cranial and spinal irradiation, which led to improvement in her pain, paresthesias, and intractable nausea.

During her hospitalization, a CT scan of the thorax incidentally revealed a pericardial effusion with tamponade physiology. A pericardial drain was placed, and fluid analysis showed CD138-positive plasma cells. Given the extent of her disease and her deteriorating condition, the patient and her family decided to transition to hospice care.

Case two

**Figure 3:**
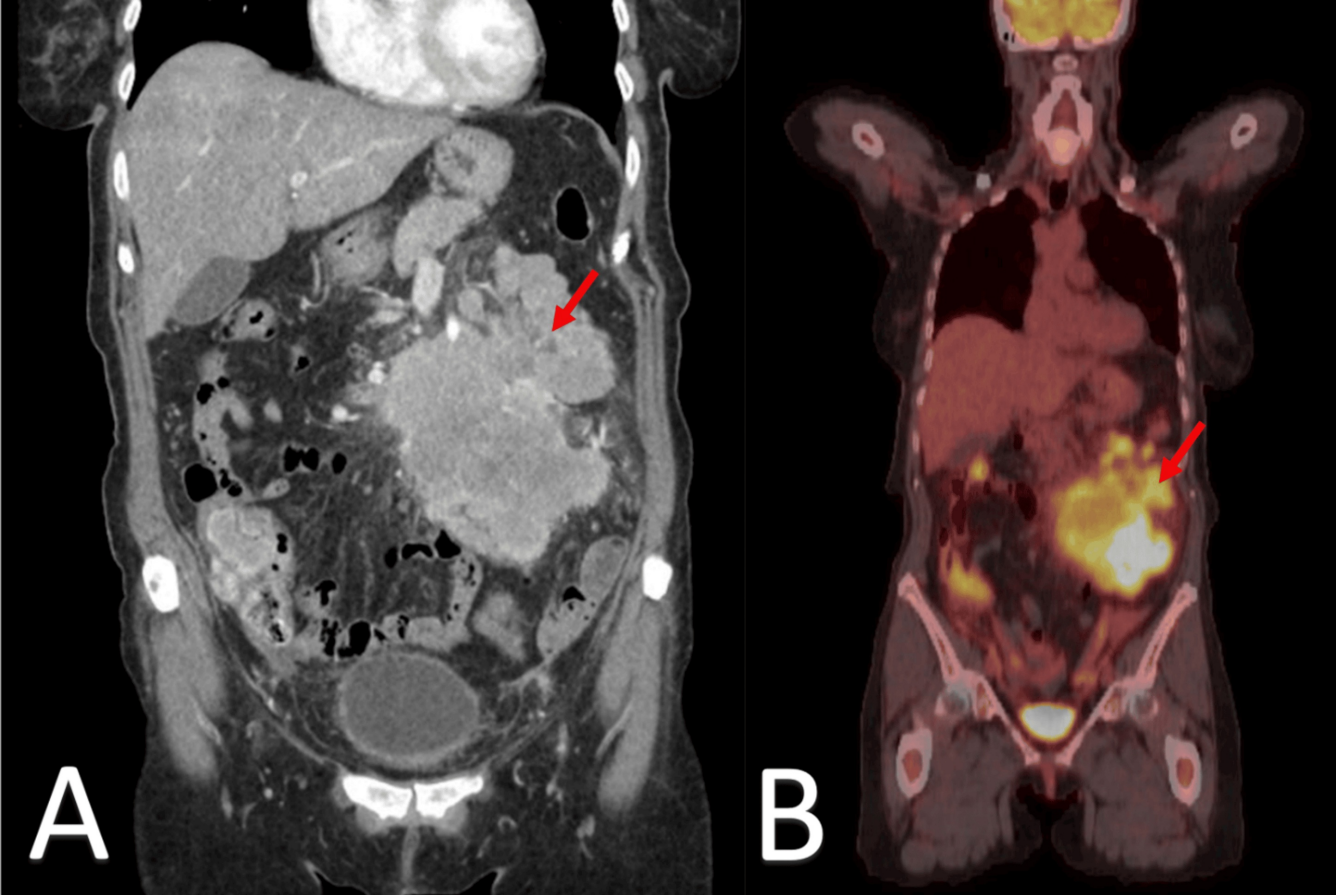
Coronal CT and PET imaging of multiple enhancing masses in the left abdomen Panel A: Coronal CT scan of the abdomen and pelvis showing a conglomerate of multiple enhancing masses (red arrows) located in the left abdomen. Panel B: Coronal PET scan highlighting the same conglomerate of masses (red arrows) with increased metabolic activity, consistent with enhancing lesions. Both imaging modalities demonstrate the presence and localization of the masses in the left abdominal region.

Our second case involves a 73-year-old female who was admitted to the hospital in November 2019 due to abdominal pain and weight loss. A CT of the abdomen and pelvis, along with a PET/CT scan, revealed a conglomerate of multiple enhancing masses in the left abdomen measuring 14 cm (Figure 3), associated with cecal wall thickening, an osseous lytic lesion in the lower back, and hypodense lesions in the liver. A biopsy of the lumbar spinous lesion showed plasma cells, kappa light chain restricted, and positive staining for CD38, CD138, CD56, and CD117. A biopsy of the mesenteric abdominal mass was consistent with plasma cell myeloma with plasmablastic features, Ki-67 80-90%, and kappa light chain restriction.

**Figure 4:**
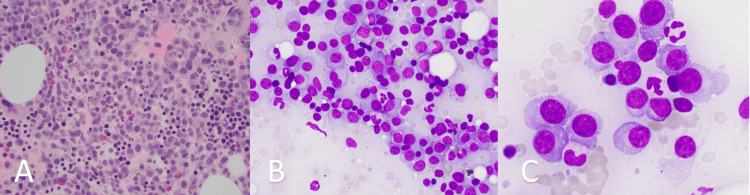
Bone marrow infiltration by plasma cells with plasmablastic features Panels A-C: Bone marrow showing effacement with plasma cells exhibiting plasmablastic features at low, intermediate, and high magnifications, respectively.

MM labs revealed an M-spike of 3.6 g/dl on serum protein electrophoresis (SPEP), with IFE showing an IgA kappa light chain phenotype. Other notable laboratory values are included in Table 1. A bone marrow biopsy revealed hypercellular marrow with marked atypical plasmacytosis (50%), consistent with, as seen in Figure 4. Flow cytometry showed CD56-/CD117-/CD138+ and CD38+ plasma cells accounting for 6-8% of all analyzed cells, with cytoplasmic kappa light chain restriction. Cytogenetics revealed a complex karyotype, with FISH results in Table 2. The patient was classified as Revised International Staging System (R-ISS) stage III.

She underwent six cycles of bortezomib, lenalidomide, and dexamethasone (VDR) followed by an autologous stem cell transplant (auto-HSCT) with high-dose melphalan (200 mg/m²) in July 2020. Following the transplant, she was maintained on lenalidomide therapy from November 2020 to April 2022. Nearly three years after her initial diagnosis, her disease recurred with the appearance of new thoracic lesions on MRI of the spine. She underwent palliative radiation and started systemic therapy with ixazomib and dexamethasone.

**Figure 5:**
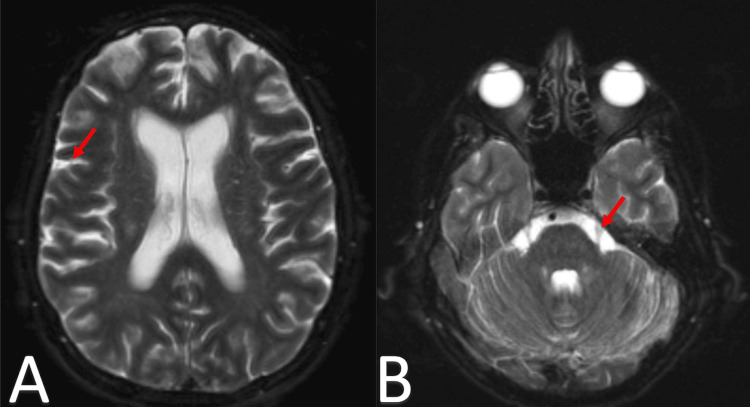
Axial brain MRI in T2-weighted FLAIR sequence demonstrating leptomeningeal enhancement and hyperintensity Panel A: Axial T2-weighted fluid attenuated inversion recovery (FLAIR) MRI showing extensive abnormal hyperintense signal and leptomeningeal enhancement in the supratentorial compartment (red arrow), raising concern for leptomeningeal involvement. Panel B: Axial T2-weighted FLAIR MRI of the infratentorial compartment exhibiting similar extensive abnormal hyperintense signal and leptomeningeal enhancement (red arrow), further suggesting leptomeningeal disease.

Two months later, while on therapy, the patient was admitted for altered mentation and seizures. An MRI of the brain revealed multiple small calvarial lesions, along with extensive abnormal FLAIR hyperintense signal and leptomeningeal enhancement throughout the infratentorial and supratentorial compartments, raising concern for leptomeningeal involvement, as seen in Figure 5. A lumbar puncture showed an elevated protein level of 194 mg/dl, with 163 nucleated cells, 98% of which were large atypical plasmablastic cells. Given her deteriorating condition, the patient's family chose to transition to hospice care.

Case three

**Figure 6:**
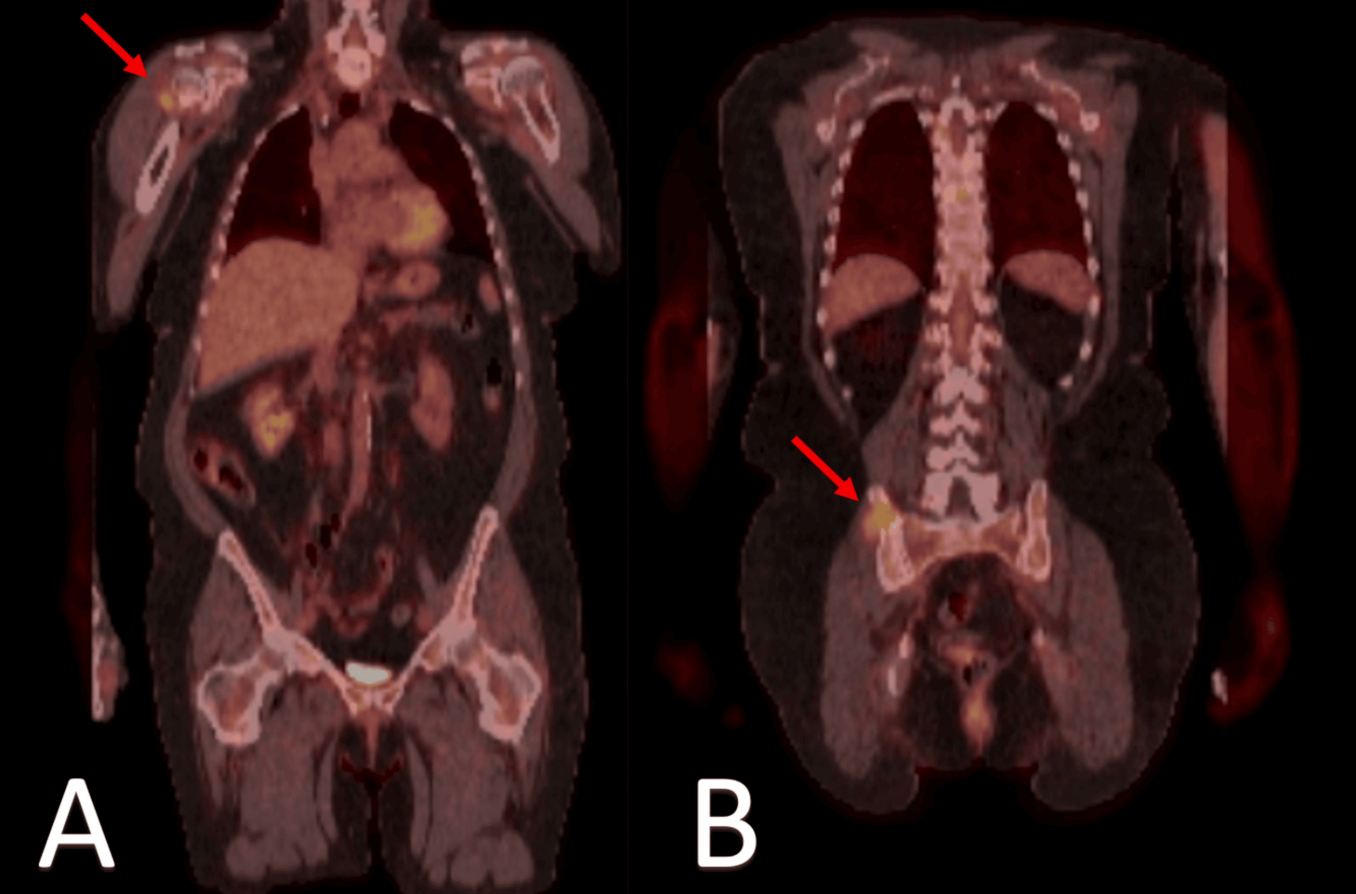
PET scan imaging of hypermetabolic osseous lesions Panel A: Positron emission tomography (PET) scan showing a lucent lesion in the right head of the humerus, indicated by the arrow, with a standardized uptake value (SUV) max of 4.0. Panel B: PET scan showing a lucent lesion in the right iliac bone (red arrow), depicting a lytic lesion with an SUV max of 5.1.

Our final case involves a 63-year-old female who presented in January 2023 with right shoulder pain and a six-month history of 30 lbs of weight loss. An MRI of the right shoulder revealed lesions in the right proximal humerus, and a PET/CT scan showed additional lesions in the right iliac bone (Figure 6). Serum protein electrophoresis with immunofixation indicated an IgA lambda light chain restricted monoclonal gammopathy. Other laboratory values are listed in Table 1.

**Figure 7:**
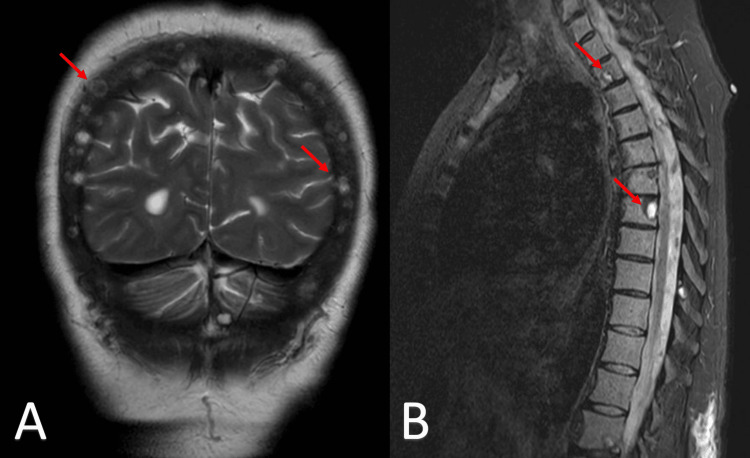
MRI imaging of osseous lesions in the calvarium and spine consistent with multiple myeloma Panel A: T2-weighted coronal MRI scan of the brain displaying osseous lesions in the calvarium (red arrows). Panel B: T2/Short Tau Inversion Recovery (T2/STIR) sagittal MRI scan of the spine showing spinal lesions at the cervical and thoracic levels (red arrows).

The patient underwent six cycles of daratumumab, bortezomib, lenalidomide, and dexamethasone (VRD), with lenalidomide held after cycle 3 due to a grade 3 rash. She also received radiation to her right arm and had internal fixation of her right humerus and left femur. In July 2023, she was referred for a transplant. Shortly thereafter, she was admitted for binocular diplopia. An MRI of the brain revealed multiple osseous calvarial lesions, but no intraparenchymal abnormalities. An MRI of the spine also showed multiple cervical, thoracic, and lumbar osseous lesions (Figure 7).** **

**Figure 8:**
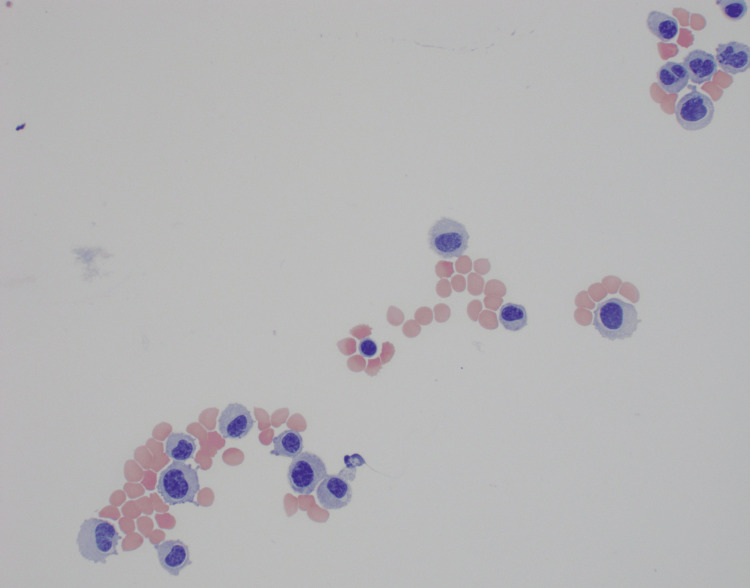
CSF cytology showing atypical plasma cells CSF: Ceebrospinal fluid.

Her symptoms were initially thought to be secondary to a transient ischemic attack (TIA), and she was discharged. However, in November 2023, she was readmitted for worsening vision, right eyelid droop, and right upper extremity weakness. An MRI of the brain showed new enhancing ovoid masses in the bilateral temporal muscles and abnormal leptomeningeal enhancement along the bilateral internal auditory canals, trigeminal nerves, oculomotor nerves, and extending into the infratemporal fossa, and bilateral foramen rotundum. A lumbar puncture showed abundant atypical plasma cells (Figure 8).

She underwent resection of a left temporal mass, which confirmed a plasma cell neoplasm. The tissue was sent for BRAFV600E mutation testing, which was negative. FISH testing did not show any translocations, and t(11;14) could not be assessed due to insufficient cellularity. Immunohistochemistry staining for cyclin D1 was positive. The patient had an Omaya reservoir placed and started twice-weekly intrathecal chemotherapy with cytarabine alternating with methotrexate. However, radiation oncology deemed craniospinal radiation too toxic to administer concurrently with intrathecal chemotherapy. Concurrently, she was started on pomalidomide, dexamethasone, and venetoclax due to her positive cyclin D1 status. Her treatment course was complicated by prolonged cytopenias, likely related to venetoclax, and her therapy was then adjusted to include only daratumumab and pomalidomide. Afterward, her cerebrospinal fluid (CSF) cleared, and she was switched to intrathecal chemotherapy every 28 days.

In February 2024, she underwent an autologous hematopoietic stem cell transplant (auto-HSCT) with conditioning using thiotepa and melphalan. Post-transplant, she continued daratumumab and pomalidomide therapy and was doing well initially. However, seven months post-transplant, her disease progressed, as evidenced by rising lambda light chains and hypercalcemia (16.5 mg/dl), which was treated with fluids, calcitonin, and pamidronate. A brain MRI at that time showed patchy meningeal enhancement. CSF analysis was inconclusive due to insufficient cellularity. She underwent intrathecal chemotherapy, and a bone marrow biopsy revealed sheets of lambda-restricted plasma cells (~50% of overall cellularity). The MM panel confirmed t(11;14) CCDN1-IGH1 mutation, while BRAFV600E mutation testing was negative. A positron emission tomography/computed tomography (PET/CT) scan revealed diffuse osseous lesions. The patient was started on venetoclax, and plans were made to initiate carfilzomib, pending insurance authorization. Ultimately, the plan was to refer her for chimeric antigen receptor (CAR) T-cell therapy.

However, she was readmitted with concerns for rapid disease progression and was treated with salvage therapy consisting of cyclophosphamide, etoposide, and dexamethasone. Flow cytometry of her peripheral blood showed 80% plasma cells, raising suspicion for plasma cell leukemia. Unfortunately, her hospital course was further complicated by E. coli bacteremia, leading to multiorgan failure and requiring pressor support. She was transitioned to hospice care and passed away eight months post-transplant.

## Discussion

Incidence and prognostic factors 

CNS-MM is rare, occurring in approximately 1% of patients. At MD Anderson at Cooper Cancer Center, we analyzed data from the last 10 years on the 284 MM patients seen in our clinic. In addition to the three above-mentioned, three more patients developed CNS involvement. This brings our institution's incidence rate of MM-related CNS involvement to 2.11%.

OS for patients with CNS-MM is significantly shorter than for those without CNS involvement, with a median survival of less than four years [7]. Key prognostic factors that impact survival and treatment response include elevated lactate dehydrogenase (LDH) levels, EMD, and high-risk cytogenetics such as t(4;14), t(14;16), and del(17p) [7,8]. Patient 1 and Patient 2 above both had t(4;14) and elevated LDH levels. Interestingly, our third case did not exhibit any of the high-risk features. In fact, she had t(11;14), which places the cyclin D1 gene under the control of the immunoglobulin heavy-chain gene, resulting in tumor cell proliferation. This is considered a moderate-risk translocation and is not typically indicative of a worse prognosis. However, this translocation has been observed to be enriched in primary Plasma Cell Leukemia, and its role in CNS-MM remains unclear [8]. Some studies suggest that the increasing incidence of EMD in recent years may be linked to the rise in autologous and allogeneic stem cell transplants [9]. However, this connection remains controversial. For instance, Pérez-Simón et al. reported a 37% incidence of EMD relapse after reduced-intensity allogeneic transplants [10], though this might reflect the underlying risk factors in patients selected for such treatments, rather than the transplant itself. In fact, the subset of patients undergoing an allogenic transplant is often patients with poor risk factors and/or aggressive disease course, both of which are associated with higher EMD risk independent of transplant. 

Symptoms

Central nervous system (CNS) involvement in multiple myeloma (MM) is a rare but severe complication that significantly impacts prognosis. Symptoms of CNS-MM are diverse and can include headaches, cranial nerve palsies (e.g., diplopia, ptosis), seizures, mental status changes (e.g., confusion, lethargy), and motor or sensory deficits (e.g., weakness, numbness). Leptomeningeal infiltration, which is common in CNS-MM, can present with symptoms such as meningismus and radicular pain. These neurological manifestations can be challenging to diagnose due to their overlap with other diagnoses [3]. The variety of symptoms observed in CNS-MM is reflected in our three cases. Patient 1 presented with nausea and vomiting, Patient 2 experienced altered mentation and seizures, and Patient 3 had vision problems, specifically binocular diplopia.

Diagnosis

Diagnosing CNS-MM involves both radiological and cytological evidence of CNS involvement. Brain and spinal MRIs may reveal leptomeningeal enhancement, intraparenchymal lesions, or spinal cord lesions. Cytological analysis of CSF often shows atypical plasma cells, with flow cytometry revealing CD38/CD138-positive cells [5].

Treatment

Currently, there is no standardized treatment for leptomeningeal involvement in MM. Several approaches are used, including systemic therapy, monoclonal antibodies, intrathecal therapy, stem cell transplantation, and radiation therapy. Each treatment option has its own limitations. CNS-MM presents a unique challenge for systemic therapy, as the treatment must be able to cross the blood-brain barrier, target the brain microenvironment, and be effective against the tumor. A retrospective study by Sammartano et al. demonstrated that systemic therapy significantly improves OS in patients with CNS-MM compared to those who do not receive it [11]. However, standard cytotoxic agents used in MM, like cyclophosphamide and melphalan, are unable to cross the BBB. Bendamustine, on the other hand, has shown some efficacy when combined with other agents [3].

Immunomodulatory drugs (IMiDs) such as thalidomide, lenalidomide, and pomalidomide have also been effective in many studies [12,13]. While proteasome inhibitors (PIs) like bortezomib are typically thought to be impermeable to the BBB, marizomib, a third-generation irreversible PI, has shown promise in glioma treatment and could be effective for CNS-MM as well [14,15]. In practice, the combination regimens, such as bortezomib-melphalan-prednisone (VMP) or lenalidomide-bortezomib-dexamethasone (RVD), with the addition of a CD38-targeting monoclonal antibody like daratumumab, are often preferred for patients ineligible for autologous stem cell transplant (ASCT) [1].

Patients with EMD tend to have higher relapse rates, so lymphoma-like chemotherapy regimens such as cisplatin-doxorubicin-cyclophosphamide-etoposide (PACE) or Dexa-BEAM (dexamethasone, carmustine, etoposide, cytarabine, and melphalan) are sometimes employed. Other regimens like cyclophosphamide-vincristine-doxorubicin-dexamethasone, alternating with methotrexate-cytarabine (HYPERCVAD), followed by ASCT or auto-allo-SCT, have also demonstrated efficacy. There is evidence supporting the role of transplant in treating CNS-MM. A study of 18 patients with CNS-MM found that the longest survivor (25 months) had undergone allogeneic stem cell transplantation (allo-ASCT), suggesting a graft-versus-myeloma effect in the CNS [3]. This was mirrored in our own experience with a patient who survived for seven months without disease progression following allo-SCT.

For patients with CNS-MM, craniospinal radiation and intrathecal chemotherapy (including glucocorticoids, methotrexate, and cytarabine) are also commonly used, often in conjunction with systemic therapy [16]. Novel agents are also being explored for CNS-MM. Isatuximab and elotuzumab, for example, are monoclonal antibodies that target specific pathways to activate immune cells. Elotuzumab works by activating natural killer cells via the SLAMF7 pathway and Fc receptors. Venetoclax, a BCL2 inhibitor, has been shown to penetrate the cerebrospinal fluid and is particularly useful in patients with t(11;22), as seen in one of our cases.

In addition, about 10% of MM patients present with a BRAF mutation at diagnosis, and up to 20% of relapsed cases show this mutation. Although there is limited data on the use of vemurafenib in relapsed MM, it may be able to cross the blood-brain barrier (BBB), as evidenced by a case report where a patient with CNS-MM responded to a BRAF-MEK inhibitor [3].

The search for new treatments is ongoing. Promising strategies include toxin-conjugated monoclonal antibodies, bispecific antibodies, and CAR-T cell therapies. For example, belantamab mafodotin, a conjugated monoclonal antibody targeting B cell maturation antigen (BCMA), has shown limited effectiveness in advanced myeloma, while teclistamab, a T-cell-redirecting bispecific antibody, has reduced efficacy in patients with plasmacytomas. However, CAR-T cell therapy has shown better outcomes in patients with plasmacytomas, demonstrating similar response rates as seen in patients without CNS involvement [17]. Whether these findings apply to CNS-MM remains unclear, highlighting the need for further investigation.

To better understand and manage CNS-MM, there is a growing consensus about the need for an international registry. As most current treatments are derived from case reports and retrospective studies, such a registry would help centralize data and improve access to the latest information [3].

## Conclusions

CNS-MM presents significant challenges in diagnosis and treatment due to its rarity and the limited availability of standardized protocols. These three cases, presented in this article, are some of the many other published cases highlighting the complex nature of this condition and underscoring the urgent need for further research to establish effective treatment strategies. As the overall survival of patients with multiple myeloma is improving, we can only expect that the incidence rate of relapses with CNS involvement will rise. As a result, it is critical to explore novel therapies and refine response criteria to enhance patient outcomes.
